# An *In Vitro* Model for the Ocular Surface and Tear Film System

**DOI:** 10.1038/s41598-017-06369-8

**Published:** 2017-07-21

**Authors:** Qiaozhi Lu, Hongbo Yin, Michael P. Grant, Jennifer H. Elisseeff

**Affiliations:** 1 Translational Tissue Engineering Center, Wilmer Eye Institute, Johns Hopkins School of Medicine, Baltimore, MD 21231 USA; 2 Department of Materials Science and Engineering, Johns Hopkins University, Baltimore, MD 21218 USA; 3 Department of Ophthalmology, West China Hospital, Sichuan University, Chengdu, 610041 China; 4 Oculoplastics Division, Ocular and Orbital Trauma Center, Wilmer Eye Institute, Johns Hopkins School of Medicine, Baltimore, MD 21287 USA; 5 Division of Plastic and Reconstructive Surgery, Shock Trauma Center, University of Maryland School of Medicine, Baltimore, MD 21201 USA; 6 Department of Biomedical Engineering, Johns Hopkins University, Baltimore, MD 21231 USA

## Abstract

Dry eye is a complicated ocular surface disease whose exact pathogenesis is not yet fully understood. For the therapeutic evaluation and pathogenesis study of dry eye, we established an *in vitro* three-dimensional (3D) coculture model for the ocular surface. It is composed of rabbit conjunctival epithelium and lacrimal gland cell spheroids, and recapitulates the aqueous and mucin layers of the tear film. We first investigated the culture conditions for both cell types to optimize their secretory functions, by employing goblet cell enrichment, air-lifting culture, and 3D spheroid formation techniques. The coculture of the two cell components leads to elevated secretion and higher expression of tear secretory markers. We also compared several coculture systems, and found that direct cell contact between the two cell types significantly increased tear secretion. Inflammation was induced to mimic dry eye disease in the coculture model system. Our results showed that the coculture system provides a more physiologically relevant therapeutic response compared to monocultures. Our work provides a complex 3D model as a recapitulation of the ocular surface and tear film system, which can be further developed as a model for dry eye disease and therapeutic evaluation.

## Introduction

The ocular surface is the outermost layer of the eye. Together with the tear film, it protects the refractive surface and enables sharp vision^1^. The ocular surface and tear film system is comprised of corneal and conjunctival epithelia, as well as many tear-secreting glands, such as lacrimal and meibomian glands^2^. They are functionally linked as one system by continuous epithelia, innervation, and the immune system^3^. The tear film is divided into three layers: mucin, aqueous, and lipids. It lubricates the eye surface, protects it against foreign pathogens and infections, and closely interacts with ocular surface epithelial cells through the mucin-aqueous layers^4^. The ocular surface and tear film system is a highly dynamic structure. The maintenance of its stability is essential to healthy vision. Even minor disruptions can result in significant permanent damage to other parts of the visual-sensory system^1^.

Dry eye is a common ocular surface disease involving dysfunction of the tear film that affects millions of people worldwide, with a significant impact on the quality of life^2^. Although the exact pathogenesis is not completely understood, it is widely believed that the development and progression of dry eye is mediated by cellular inflammatory molecules secreted by the ocular surface immune system^5^. When the ocular surface is exposed to environmental stress that causes changes in tear composition, an inflammatory cascade is activated in which various cytokines and chemokines are released^6^. This results in the migration of antigen presenting cells, and subsequently the infiltration of helper T cells (subtypes T_H_1 and T_H_17) to the ocular surface. During this time more cytokines and chemokines are secreted, and the epithelia are consequently damaged^5, 6^. To learn more about the molecular basis of dry eye, disease pathogenesis study and potential therapeutic evaluation have been conducted either *in vivo* or on simple models *in vitro* ^7, 8^.

Tissue models and organ-on-a-chip strategies combine multiple cell types to create miniaturized versions of physiologically active and functional tissues in the body. Numerous organ systems, such as lung, intestine, and bone marrow, have been successfully fabricated and utilized in various disease conditions^9–11^. Specifically, a complex disease system was induced in a lung-on-a-chip microdevice, leading to the discovery of a potential new drug for pulmonary edema^12^. Numerous *in vitro* models have been established in the past few years to mimic different parts of the ocular system. Mature retina was generated from human induced pluripotent stem cells (hiPSCs) with fully functional photoreceptors to recapitulate retinal development. This system was applied to model retinal dysfunctions, including age-related macular degeneration^13^. Coculture of retinal pigment epithelium (RPE) and photoreceptors has been attempted to further mimic normal differentiation and morphology^14^. Moreover, Chan KY, *et al*. manufactured a microfluidic chip to study the intraocular emulsification of silicone oil^15^. An *in vitro* corneal model was developed on a sacrificial collagen film across microfluidic channels, presenting an alternative for the screening of ocular irritants^16^.

**Figure 1:**
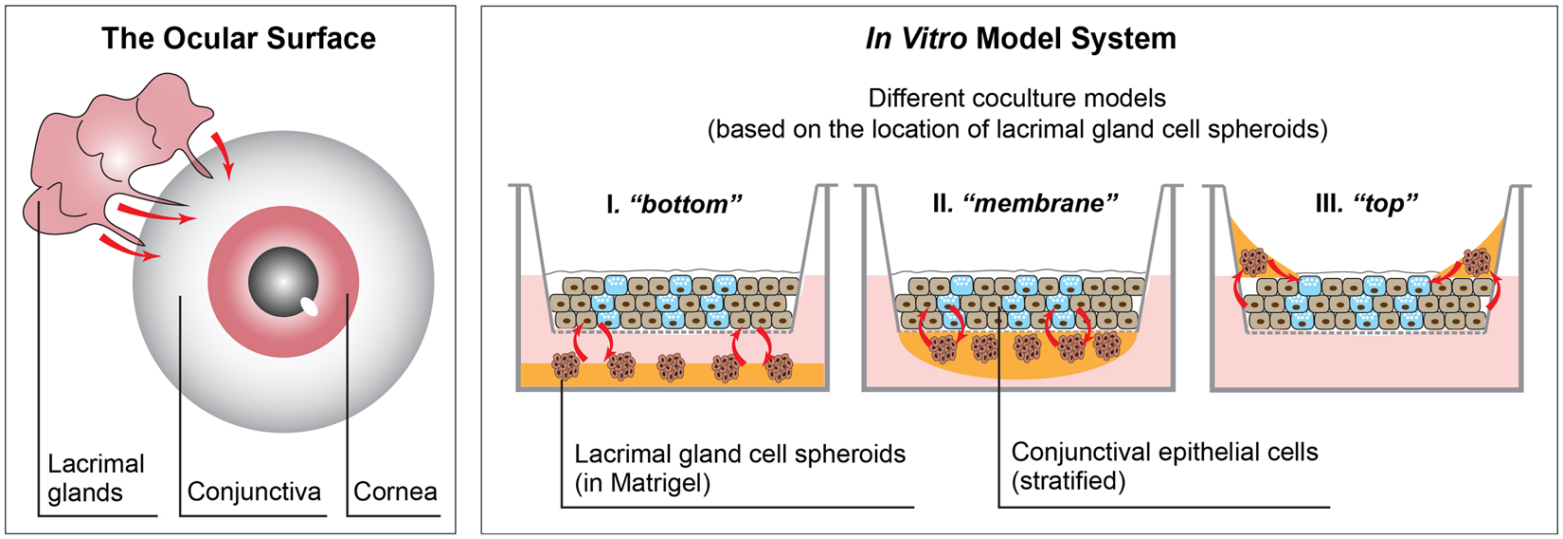
The ocular surface and *in vitro* model system, showing models with different cell organizations. The name of coculture models were based on the location of LG cell spheroids. Red arrows indicate direct or indirect interactions between different components.

Considering the ocular surface and tear film, researchers have been investigating the *in vitro* modeling of several components individually, including conjunctival epithelium^17^ and lacrimal glands^18, 19^. In these models, cell morphology and phenotype resembled the equivalent cells *in vivo*. However, combining these cells types to create a functional tear film has not been studied. To address this gap, we developed a complex three-dimensional (3D) *in vitro* model for the ocular surface. This model contains primary rabbit conjunctival epithelial cells (CECs) and lacrimal gland (LG) cell spheroids, to recapitulate the aqueous and mucin layers of the tear film. As a discrete exocrine gland, the lacrimal glands are not in close proximity with conjunctiva or cornea (Fig. 1). However all components of the ocular surface are closely integrated together, and one component can have substantial influence over the secretory function of another in this system^3^. In our model system, we introduced direct or indirect cell-cell contact between the two cell types, and studied the influence on secretory functions in three different coculture models (Fig. 1). We first investigated the culture conditions for both cell types to optimize their secretory functions. Next, we combined the two cell types and compared several coculture systems to optimize tissue structure and tear secretion. Inflammation was induced to mimic dry eye disease in the coculture model system, and its response to therapeutics was compared to monocultures. Overall, we engineered a model system for both a healthy and diseased ocular surface amenable to studying disease pathogenesis and therapeutic screening.

## Results

### Goblet cell enrichment and air-lifting culture of CECs

**Figure 2:**
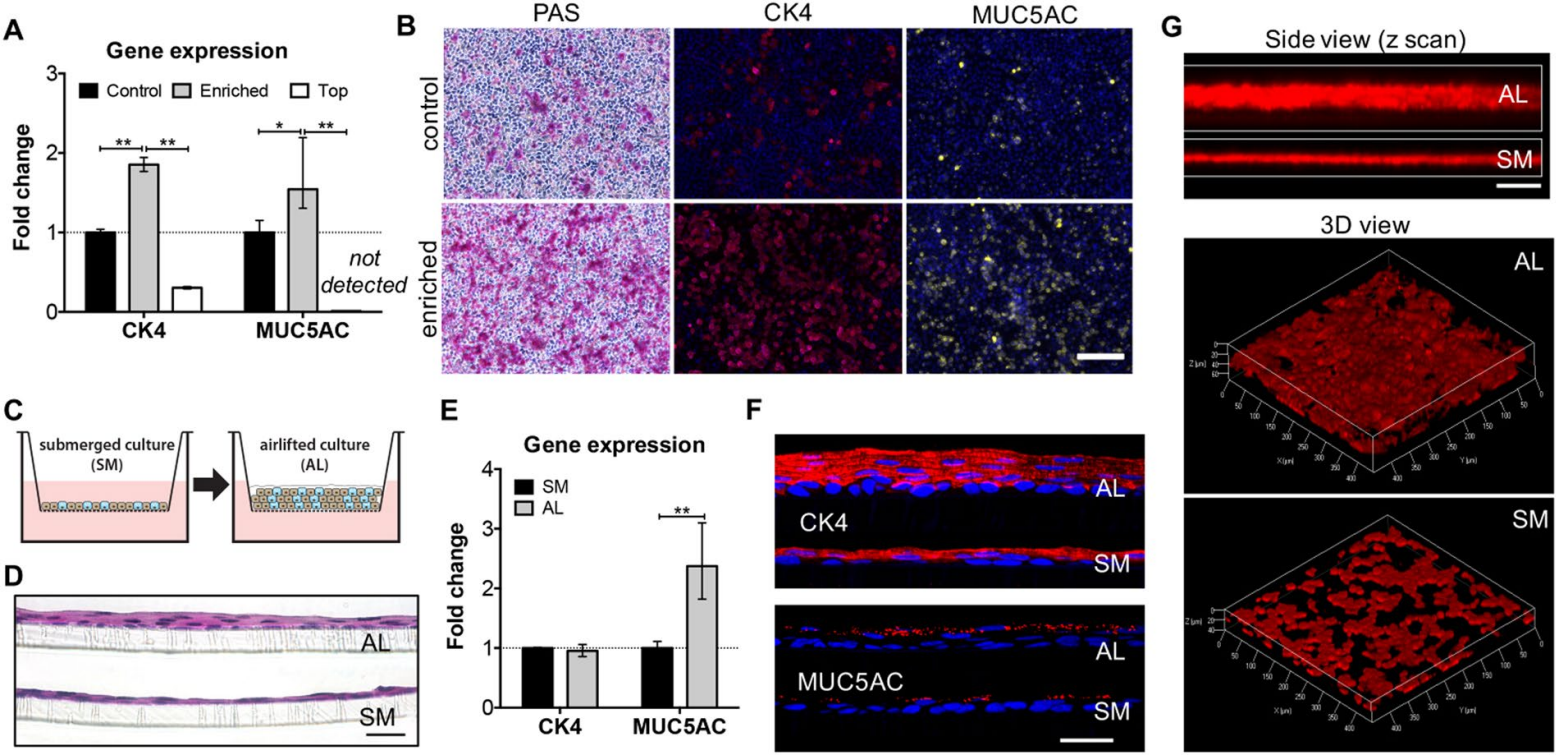
Goblet cell enrichment and airlifting culture of conjunctival epithelial cells (CECs). (**A**,**B**) Using Percoll density gradient to increase the initial goblet cell percentage in primary conjunctival epithelial cell populations. (**A**) Gene expression (RT-qPCR; fold change is expressed as 2^−ΔΔCT^) of CECs before (control) and after goblet cell enrichment. CECs were separated into two populations after Percoll gradient separation – top and bottom (enriched). (**B**) PAS staining and immunohistochemistry (IHC) for CK4 and MUC5AC comparing the phenotypes of CECs with or without goblet cell enrichment. Scale bar 200 μm. (**C**) The schematics for airlifting (AL) and submerged (SM) cultures of CECs based on Transwell insert. H&E staining (**D**) and RT-qPCR (**E**) comparing CECs under AL and SM culture. Scale bar 100 μm. (**F**) IHC (top: CK4; bottom: MUC5AC) showing the difference in cell phenotypes between AL and SM cultures. Scale bar 50 μm (**G**) Mucin secretion (stained by Texas Red labeled dextran) visualized by confocal microscope (z scan). Both side view and 3D views are shown. Scale bar 100 μm. *p < 0.05; **p < 0.001.

Primary rabbit CECs were isolated from rabbit conjunctival tissues. To increase the initial percentage of goblet cells within the primary epithelial cell populations and better represent the physiological condition, CECs were subject to a Percoll density gradient where six different layers were tested (Fig. S1A; see supplementary information). Cells in layers 1 and 2 expressed significantly lower levels of *cytokeratin 4* (*CK4*) and *mucin 5AC* (*MUC5AC*) compared to the cell population before Percoll separation (Fig. S1B). Therefore, 30% Percoll/PBS solution was selected for goblet cell enrichment for all subsequent studies, and the three cell populations were named as top, bottom (enriched), and control (unseparated) (Fig. S1A). In general, the enriched CECs (with increased percentage of goblet cells) expressed significantly more *CK4* and *MUC5AC* mRNAs than the control, while the top layer had much less expression of *CK4* (<0.3-fold), and expression of *MUC5AC* was undetectable (Fig. 2A). Enriched CECs also expressed more epithelial markers in culture and secreted more mucosubstance when compared to unseparated control, as confirmed by CK4, Periodic acid-Schiff (PAS), and MUC5AC stainings, respectively (Fig. 2B).

Airlifting culture was compared to submerged culture to optimize the mucin secretion by CECs *in vitro* (Fig. 2C). After one week of airlifting, CECs stratified into 5–6 layers of epithelial cells, while submerged culture had less than two layers of cells (Fig. 2D). Immunohistochemistry for CK4 and MUC5AC (Fig. 2F) confirmed improved epithelial and goblet cell differentiation as well. Airlifting also significantly benefited the mucin secretion of CECs. Three-dimensional confocal *z*-scanning of the mucin layer, stained by Texas Red-dextran, confirmed that airlifting resulted in a thicker and more continuous extracellular mucin secretion (Fig. 2G). The side view of the confocal *z*-scanning indicated an overall thicker mucin layer (50.0 ± 5.0 µm for airlifting culture and 15.0 ± 1.7 µm for submerged culture). Though *CK4* expression was the same in both cultures, airlifting culture significantly increase the mRNA level of *MUC5AC* (Fig. 2E).

### The formation and development of 3D LG cell spheroids in Matrigel

**Figure 3:**
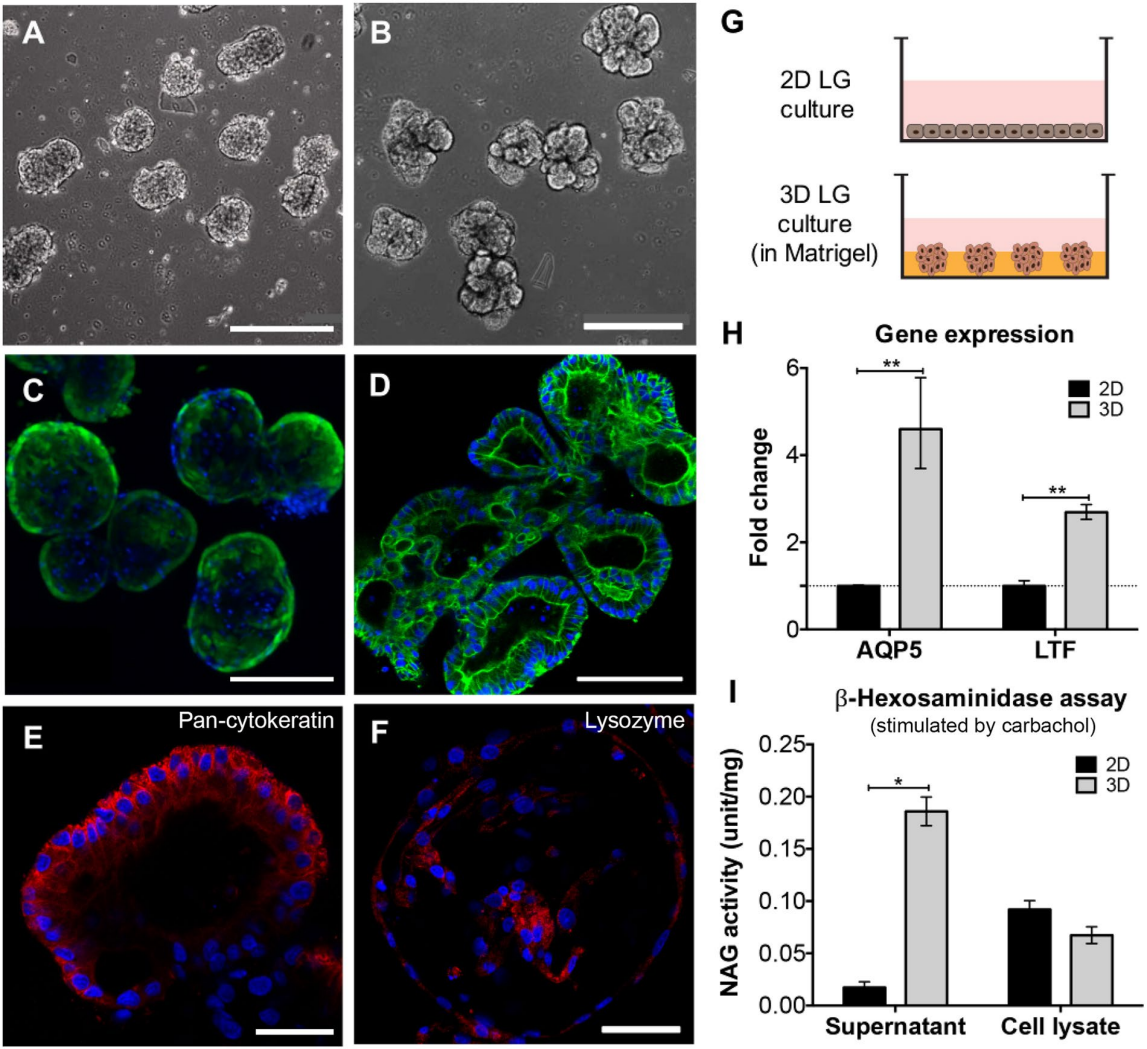
3D lacrimal gland (LG) cell spheroids formation and culture. (**A**–**D**) Phase contrast and F-actin staining (Phalloidin) images of LG cell spheroids in Matrigel at different stages. (**A**,**C**) day 2; (**B**,**D**) day 5. (**E**,**F**) IHC showing the expression of LG specific markers (red; counterstained by DAPI). (**E**) Pan-cytokeratin; (**F**) lysozyme. Scale bar: 400 μm (**A**,**B**); 200 μm (**C**); 100 μm (**D**); 50 μm (**E**,**F**). (**G**) Schematics of LG cultured as either 2D monolayer or 3D spheroids embedded in Matrigel. (**H**) Gene expression (RT-qPCR; fold change is expressed as 2^−ΔΔCT^) and (**I**) β-hexosaminidase assay comparing LG cells culture under 2D and 3D conditions. Both supernatant and cell lysate were collected for hexosaminidase assay and the value (units) was normalized to total protein content (mg). *p < 0.05; **p < 0.001.

Primary rabbit LG acinar cells (LGACs) spontaneously formed spheroids after 24 hours on the orbital shaker, and the average diameter of the spheroids was 124 ± 19 µm (Fig. S2). The spheroids underwent structural development in Matrigel to produce glandular tissue. After encapsulation, the LG cell aggregates formed spheroids at day two (Fig. 3A), and continued to develop into multi-branched organoids by day five (Fig. 3B). Phalloidin staining revealed formation of hollow cavities inside the spheroids (Fig. 3C). We also assessed the phenotype and function of the lacrimal gland organoids formed *in vitro* by evaluating several important makers, including lysozyme and lactoferrin (LTF), two enzymes in the tear film secreted by LGACs^20, 21^, and aquaporin 5 (AQP5), residing on the cell membrane of LGACs and whose level is significantly altered in dry eye disease^22^. By day 5, each spheroid further developed multiple acinus-like compartments (Fig. 3D). The LG cell spheroids stained positive for both epithelial maker pan-cytokeratin (PCK, Fig. 3E) and LYZ (Fig. 3F).

Next, the function of LG cells in 2D monolayer or 3D spheroids in Matrigel matrix was compared (Fig. 3G). Three-dimensional culture influenced tear film secretion on the transcriptional level. The genes aquaporin 5 (*AQP5*) and lactoferrin (*LTF*) increased 4- and 2.5-fold, respectively, in the 3D culture (Fig. 3H). Media supernatants and cell lysates were collected to measure the concentration of active lysozyme (β-N-Acetylglucosaminidase, NAG; measured by β-Hexosaminidase assay after 100 μM carbachol stimulation) in the tear film secreted by the lacrimal glands. The cell lysate of LG cells cultured in 2D monolayer had a higher level of active lysozyme, but the lysozyme concentration in the supernatant of 3D spheroid cultures was more than eight times than that of 2D culture (0.0017 vs 0.0002; Fig. 3I).

### Coculture of airlifted CECs and LG cell spheroids

**Figure 4:**
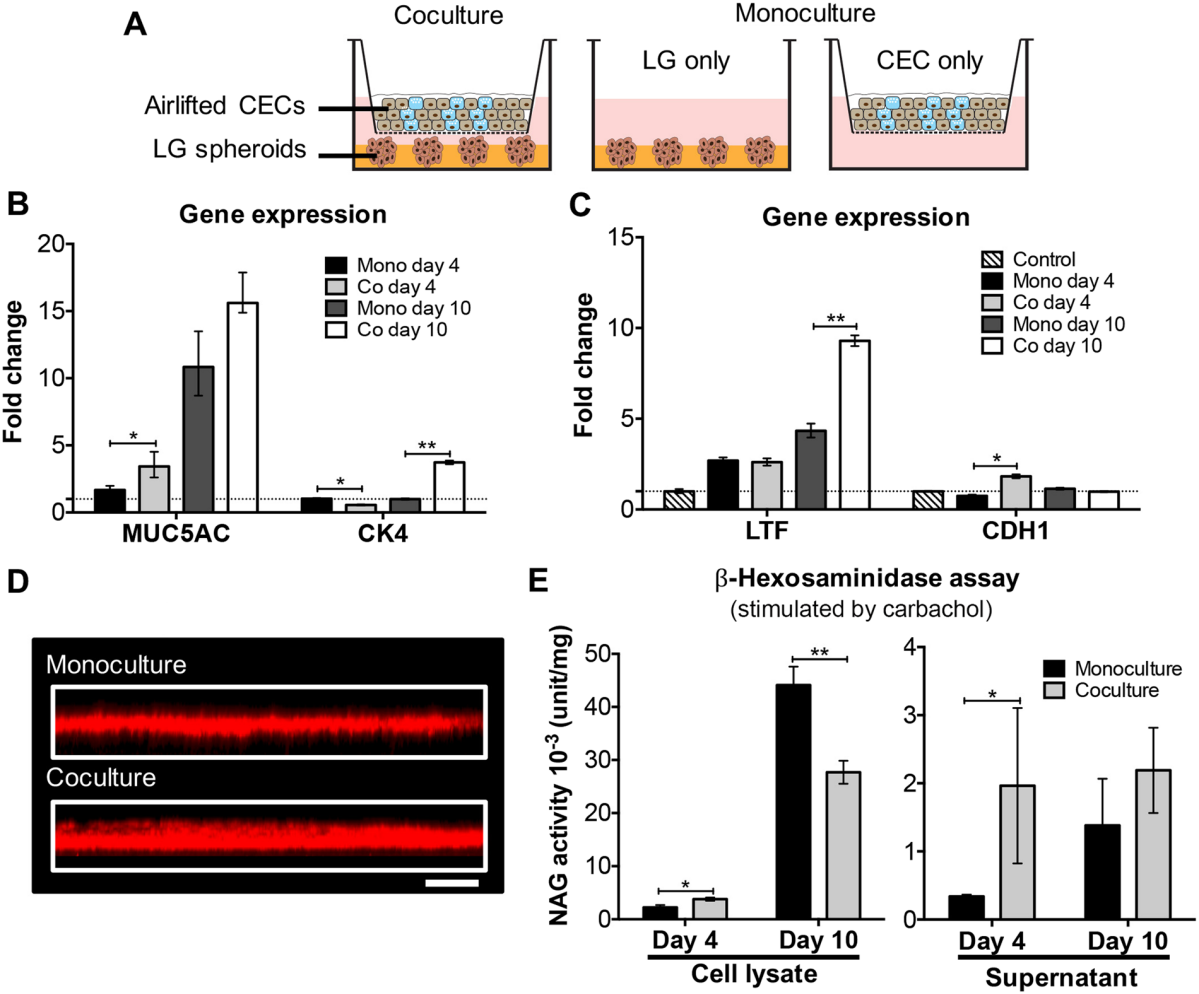
Coculture of conjunctival epithelium and lacrimal glands. (**A**) Schematics showing the setup of coculture in the Transwell system. Airlifted CECs cultured on the insert were combined with LG/Matrigel in the lower compartment to achieve coculture. In monocultures, only one cell population was present. (**B**,**C**) The difference in gene expression between monocultures and cocultures. (**B**) Conjunctival epithelium specific markers; (**C**) lacrimal gland specific markers. Samples at day 4 and 10 were collected and analyzed. Control in (**C**) was LG cells culture in 2D monolayer. (**D**) Confocal (z scan) microscopic image of the secreted mucin layer from airlifted CECs cultured in monocultures and cocultures. Scale bar 100 μm. (**E**) β-hexosaminidase assay of LG cell lysates and supernatants. Samples at day 4 and 10 were collected and analyzed. *p < 0.05; **p < 0.001.

Airlifted CECs and LG cell spheroids were cultured together to study the effect of coculture on tear secretion and gene expression. Stratified CECs were on the upper compartment and encapsulated LG cell spheroids were on the lower compartment. The two monoculture groups were composed of either LG cell spheroids, or air-lifted CECs (Fig. 4A). By day 10, air-lifted CECs under both coculture and monoculture conditions secreted a continuous layer of extracellular mucins. The average thickness of mucin film secreted by cocultured CECs was 45.8 ± 5.3 µm, as compared to 30.0 ± 5.0 secreted by monocultured CECs (Fig. 4D). The media supernatants and LG cell lysates were collected for the measurement of lysozyme concentration. At day four, cocultured LG cell spheroids secreted more than 10 times the amount of active lysozyme into the media supernatant compared to monocultured LG cell spheroids; however, this difference decreased at day 10 (Fig. 4E; after carbachol stimulation). Regarding the cell lysates, coculture was beneficial for lysozyme synthesis at day 4; however, monocultured LG cells contained more lysozyme than cocultured cells at day 10. Regarding gene expression, the cocultured group reported elevated level of E-cadherin (*CDH1*) and *MUC5AC* at day four, and *LTF* and *CK4* at day 10 (Fig. 4B,C). Gene expression values are normalized to cells cultured in monolayer.

### The influence of cell contact on the secretory function in coculture

**Figure 5:**
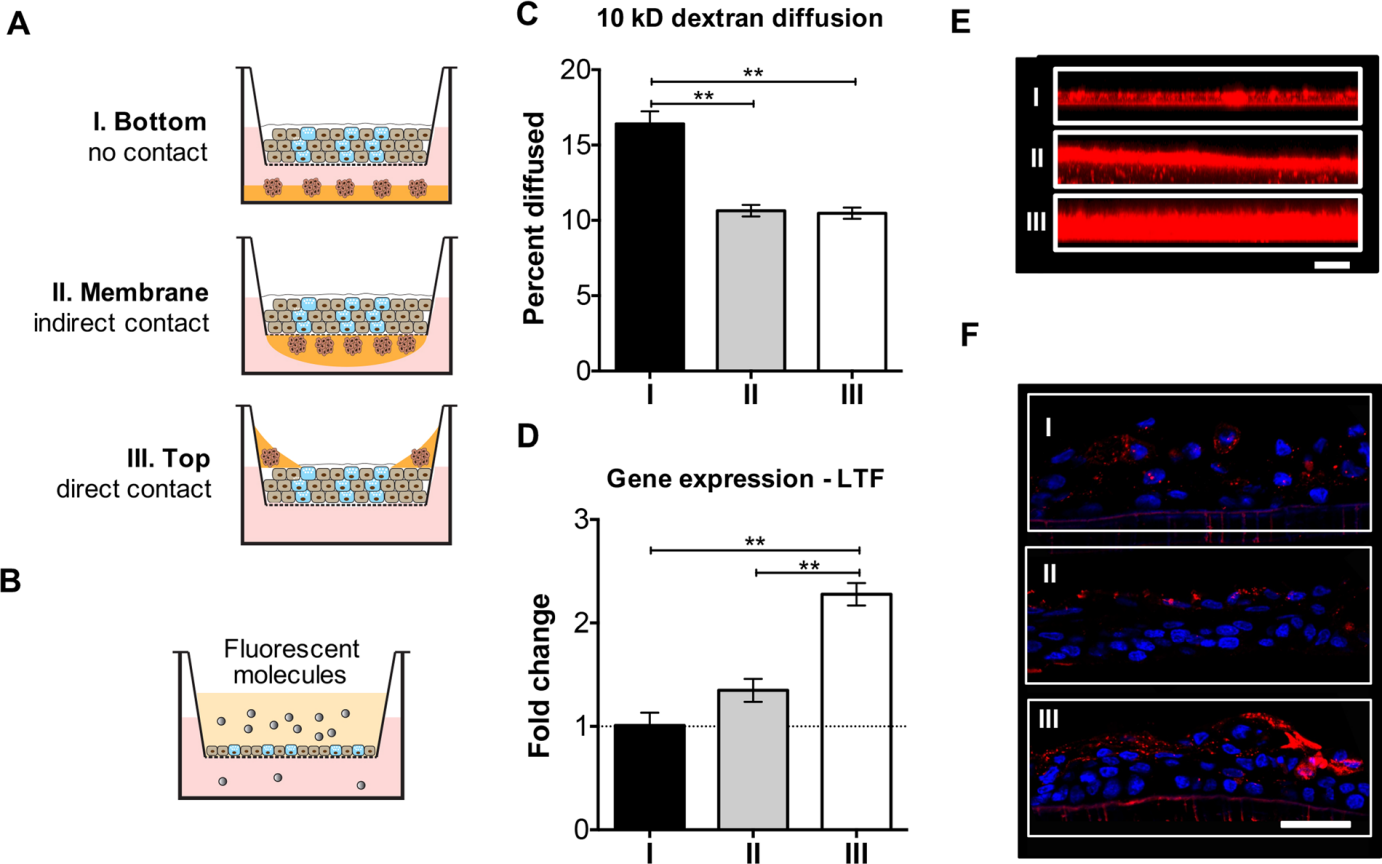
Comparison of different coculture model systems. (**A**) The three different designs of coculture system, which were named after the relative interactions between CECs and LG cell spheroids. (**B**,**C**) Diffusion study (with fluorescent 10 kD dextran). Data in (**C**) was presented as percent of dextran diffused through. (**D**) LTF gene expression across the three coculture designs. (**E**) Secreted mucin layer from airlifted CECs in coculture, visualized by confocal microscopes (z scan). Scale bar 100 μm. (**F**) MUC5AC IHC staining (red fluorescence; nuclei were counterstained with DAPI) of the airlifted CECs. Scale bar 50 μm. *p < 0.05; **p < 0.001.

Different coculture architectures were developed to investigate the influence of cell contact on the function of the *in vitro* model system. The three designs tested were based on the degree of contact between the two cell types: (I) Bottom: no cell contact, (II) Membrane: indirect cell contact (separated by a porous membrane), and (III) Top: direct cell contact (Figs 1 and 5A). Permeability test with 10 kD Texas Red-dextran (Fig. 5B,C) was conducted to assess the tight junctions of CECs in different coculture models. Less than 17% of dextran diffused through during this experiment in all three models. Moreover, when there was indirect or direct cell contact between the two cell populations, the diffusion percentage reduced to about 10%, significantly lower compared to the no cell contact model.

The level of cell contact also impacted the level of gene expression and protein secretion in the coculture models. The amount of *LTF* mRNA in the direct cell contact model was twice as much as those in the other two models (Fig. 5D). This same trend was observed in the measurement of secreted mucin layer. The average mucin thickness values for models (I–III) were 31.5 ± 1.7 µm, 40.3 ± 2.5 µm, and 82.1 ± 4.0 µm, respectively (Fig. 5E). Specifically, direct cell contact between CECs and LG cell spheroids gave rise to the highest level of MUC5AC staining compared to the other two models (Fig. 5F).

### Direct cell contact coculture system as a complex 3D dry eye model *in vitro*

**Figure 6:**
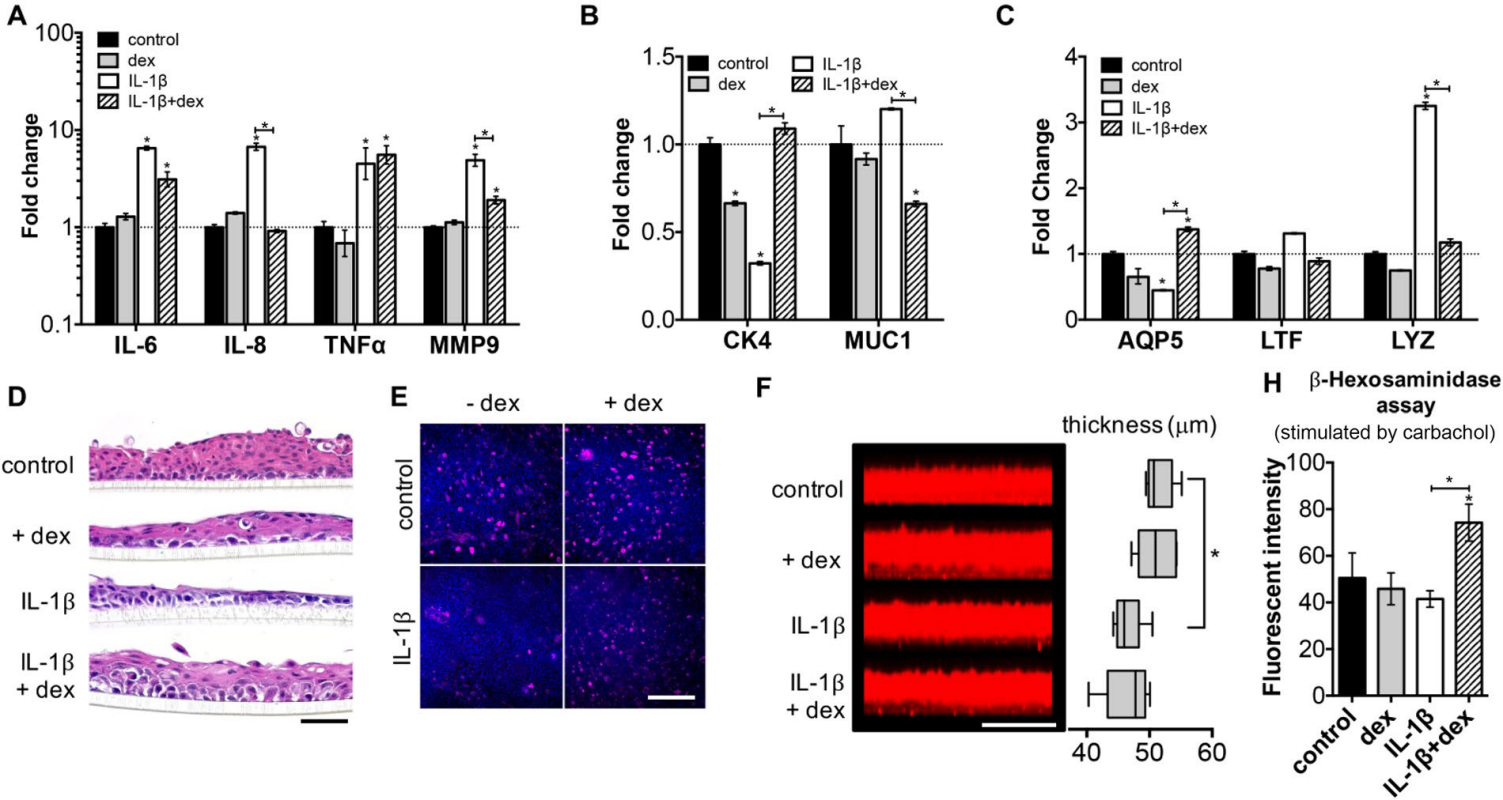
Responses of direct cell contact model after cytokine IL-1β stimulation. (**A**–**C**) Gene expression of the coculture system after the addition of IL-1β. (**A**) Inflammatory genes; (**B**) conjunctival epithelial specific genes; (**C**) lacrimal gland specific genes. (**D**) The change of epithelial thickness and morphology (H&E staining) after IL-1β exposure. Scale bar 100 μm. (**E**) MUC5AC staining of the airlifted CECs under various conditions (top view). Scale bar 200 μm. (**F**) Mucin layer secreted by airlifted CECs imaged by confocal z-scanning. The thickness values were measured using ImageJ. Scale bar 100 μm. (**H**) β-hexosaminidase assay (supernatant of LG cell spheroids culture; measured by fluorescent intensity). Dexamethasone: dexamethasone, used as a treatment to inflammatory caused by IL-1β. *p < 0.05; **p < 0.001. Significance indicates comparison with the control group if not stated otherwise.

Using the coculture system that provided the best mimic for the ocular surface and tear film, response to inflammation-inducing molecules was tested. Specifically, the direct cell contact model system was exposed to the proinflammatory cytokine interleukin 1 beta (IL-1β) and the commonly used therapeutic dexamethasone. IL-1β exposure upregulated the mRNA level of various cytokines and chemokines, including *IL-6*, *IL-8*, *tumor necrosis factor alpha* (*TNFα*), and *matrix metalloproteinase-9* (*MMP9*), at both six- and 24-hour time points (Fig. S3A and Fig. 6A). Dexamethasone was effective in downregulating these genes at the 24-hour time point (Fig. 6A). Cytokine exposure also changed the expression of ocular surface specific genes (Fig. S3B,C and Fig. 6B,C). The level of *CK4* and *AQP5* was reduced, while LYZ expression was increased upon the addition of IL-1β. When CECs or LG cell spheroids were cultured alone (Fig. S4), IL-1β also induced significant changes in gene expression. However, the addition of dexamethasone was ineffective in restoring the mRNA level of either proinflammatory or tissue-specific genes (Fig. S4), highlighting the importance of the coculture system in providing a physiologically-relevant response to therapeutics.

IL-1β exposure resulted in thinning of the conjunctival epithelial in the ocular surface model. Treatment with dexamethasone restored the thickness of epithelium after IL-1β exposure (Fig. 6D). A similar trend was observed with MUC5AC staining (Fig. 6E), in which goblet cells (MUC5AC+) were almost absent with IL-1β culture. IL-1β also significantly decreased the thickness of the tear film secreted by the model system (Fig. 6F), while the combination of IL-1β and dexamethasone increased lysozyme secretion (Fig. 6H; after carbachol stimulation).

## Discussion

Conjunctival epithelium, goblet cells, and lacrimal glands are parts of the ocular surface and tear film system. The mucin-aqueous layer in the tear film is of essential importance in protecting the ocular surface epithelium, and is frequently damaged in dry eye disease. Therefore, we constructed a model system for rabbit ocular surface in which the synergistic interactions between conjunctival epithelium and lacrimal glands can be realized and precisely examined.

Coculture is a common technology utilized in tissue engineering to study the natural and/or synthetic interaction between cell populations, and to improve *in vitro* culture time^23^. In the ocular system, coculture of RPE and photoreceptors was able to provide a better mimic of retinal differentiation and morphology^14^. Dry eye is an internationally prevalent disease with few efficacious therapies. Besides animal models, robust *in vitro* models of the ocular surface and tear film provide a tool to study pathogenesis and to improve drug screening. Thus, we cocultured conjunctival epithelial and lacrimal gland cells, two important components for tear secretion, in the ocular surface model system. The coculture system was compared to monocultures with respect to the expression of tear secretory markers. Goblet cells in coculture contributed to a thicker mucin layer, and LG cell spheroids had elevated secretion as well. Coculture also helped maintain the morphology and phenotype of the two cell populations (Fig. 4).

To create functional tissue mimics, we need to consider the effect of cell contact. Different organ/tissue systems require special consideration while designing coculture models. For example, Wallace CS *et al*. developed tissue engineered blood vessel model by coculturing endothelial (ECs) and smooth muscle cells (SMCs). In this model quiescent SMCs reduced the inflammatory response of ECs mediated by TNFα^24^. To find the most effective coculture model and analyze the secreted tear fluid, three different coculture models were compared (Figs 1 and 5). Results of the permeability test confirmed that the tight junction of conjunctival epithelium is not compromised by the introduction of cell contact in models (II) and (III). The thickness of tear fluid increased significantly with more cell contact (Fig. 5E), especially in the direct cell contact model (III). Because the stratified CECs remained as a barrier between the LG cell spheroids and the medium reservoir below, the tear fluid secreted by LG cell spheroids accumulated on the top chamber instead of diffusing into the reservoir. This resulted a mixture of both mucin and aqueous layers. This design allowed the secretion from LG cell spheroids to have direct influence on CECs, and it can also enable the extraction of the secreted tear fluid for further proteomics and rheological analyses.

Although conjunctival epithelium and lacrimal glands do not have direct contact anatomically (Fig. 1), the unique organization of the two cell populations in the direct cell contact model leads to greatly improved tear secretory function compared to other models. It provides an innovative design for the collection and analysis of secreted tear fluid. Moreover, the unique architecture supported long term culture *in vitro*. We tested this specific model system as a mimic for aqueous dry eye disease, induced by IL-1β, an early molecular mediator in the progression of inflammatory diseases^25^. After IL-1β exposure, at early time points, we observed changes in the mRNA level of various proinflammatory factors as well as tear secretory markers. At later time points, the number of goblet cells was reduced significantly, and tear secretion from both cell populations decreased when IL-1β was present. Epithelial damage and thinning were also observed, possibly due to the release of MMPs (Fig. 6). Dexamethasone is an anti-inflammatory corticosteroid, known to be beneficial in treating dry eye disease^26^, and it was able to counteract the effect of IL-1β on our model system. The outcomes we observed in the direct cell contact model system mimic moderate dry eye symptoms in animal models^27^. As a comparison, we also examined the response of monocultured CECs or LGs to cytokine and steroid treatment. Monocultured CECs and LG cell spheroids both showed altered gene expression in response to IL-1β. However, treatment with dexamethasone could not alleviate these symptoms (Fig. S4). Therefore, based on the different responses between cocultured and monocultured cells, the complex 3D coculture system is a more reliable dry eye model *in vitro* compared to monocultured models because it provides a physiologically-relevant response to treatment, and is necessary for the accurate evaluation of therapeutic effects. For further analyses and improvement of the model, humidity and air flow rate could be controlled for more accurate mimicking of dry eye disease, and rabbit primary cells could be substituted with human cells as well.

The mucin layer of the tear film is critical in maintaining homeostasis of the ocular surface. Low goblet cell density and a disrupted mucin layer could accelerate the vicious cycle in the development of dry eye^28^. Culture methods have been established for studying the biology of goblet cells and mucin secretion^29, 30^. However, previously established conjunctival epithelial models have lower percentages of goblet cells than their corresponding native tissue^31^. To ensure adequate goblet cell number and mucin secretion, CECs were subject to Percoll density separation and airlifting culture. After Percoll separation, the gene expression results indicated that the bottom cell population was enriched with *MUC5AC* positive goblet cells, as well as *CK4* positive epithelial cells. With the Percoll gradient enrichment, primary CECs were able to give rise to more goblet and epithelial cells in culture, confirmed by immunocytochemistry (Fig. 2A,B). Airlifting culture is essential for goblet cell maturation and mucin secretion, and the development of a normal mucin layer in the tear film^32^. After one week of airlifting culture, cells stratified into four to five layers of CK4 positive epithelial cells and MUC5AC producing goblet cells (Fig. 2C–F). Airlifted CECs also secreted a thicker and more homogenous mucin-aqueous thin film, labeled by fluorescent dextran (Fig. 2G).

Lacrimal glands are the main secretory source of the aqueous tear components. Aqueous deficient dry eye usually involves decreased secretion and inflammation in the lacrimal glands^2^. A 3D environment is required for the successful mimicking of this condition. To investigate the mechanism of lacrimal gland secretion and dry eye disease, various methods have been attempted to recapitulate the 3D environment *in vitro*, including rotary cell culture system^33^ and Matrigel raft culture^34^. Matrigel is prepared from the basement membrane of the EHS mouse sarcoma line, and rich in extracellular matrix molecules, including many growth factors^35^. Successful cultures of lacrimal gland acinar cells have been established on Matrigel coated plates since the 1990s^36, 37^. We employed a simple yet effective method to form homogenous LG cell spheroids by self-assembly, which were later harvested and encapsulated in Matrigel. The spheroids underwent organoids development, formed hollow cavities first, and then were interconnected together with ductal structure into multi-branched lobules with lumens. These lacrimal organoids were functionally active, confirmed by lysozyme staining (Fig. 3).

Our model system proved that coculture introduced beneficial effect on the secretory function of both CECs and LG cell spheroids, and it more closely mimicked the pathophysiology of dry eye disease. *In vivo*, secretions from the LGs are transported to conjunctival epithelium via lacrimal ducts, and this interaction was realized in our “top” coculture model. Although the conjunctiva does not have direct access to LGs, changes and disruptions initiated in the conjunctiva can still have substantial influence on LGs, because all parts of the ocular surface are integrated together via innervation and circulation. For example, desiccating stress to the cornea or conjunctiva eventually can cause changes in cytokine expression and eventually alteration in tear composition^6^. To study whether the beneficial effects of coculture is due to direct cell-cell contact or secretory factors exclusively, conditional medium can be used to replace CECs or LG cell spheroids. Furthermore, the model system can be established on a microfluidic device where the interaction between two cell types can be more precisely controlled.

In summary, we engineered a synthetic coculture model system, composed of conjunctival epithelium and LG cell spheroids, as a recapitulation for the ocular surface and aqueous tear system. We demonstrated the efficacy of this model system as a mimic for both the healthy ocular surface and aqueous dry eye disease induced by inflammation. Our model system provides a novel platform for the pathophysiological study of the ocular surface, as well as for the discovery of new therapeutics for dry eye disease.

## Materials and Methods

### Primary cell isolation

Young New Zealand white rabbit tissues (eyes and lacrimal glands) were purchased from Pel-Freez Biologicals (Rogers, AR).

#### Rabbit CECs

Rabbit eyes with eyelids attached were rinsed with DMEM/F12 (Dulbecco’s Modified Eagle Medium/Nutrient Mixture F-12; Thermo Fisher Scientific, Waltham, MA) with 1% antibiotic-antimycotic solution (Thermo Fisher Scientific). The entire conjunctiva was dissected 1 mm from the glandular edge of the tarsal plate and 2 mm from the limbus. The tissue was digested with Dispase® II (1.2 U/mL, Roche Diagnostics, Indianapolis, IN) at 4 °C overnight. The loosened epithelial aggregates were collected with a cell scraper, followed by centrifugation at 200 g for 5 minutes. The cell pellet was treated with Accutase® (Sigma-Aldrich, St Louis, MO) for 10 minutes at 37 °C and filtered through a 100 μm cell strainer to obtain a single cell suspension.

#### Rabbit LGACs

Inferior lacrimal glands from young rabbits were rinsed with DMEM/F12 (with 1% antibiotics-antimycotics) and finely minced into small pieces in a petri dish. The isolation procedure was similar as previously described^38^. Briefly, the minced tissues were digested with an enzyme cocktail containing collagenase (Worthington Biochemical, Lakewood, NJ), hyaluronidase (Sigma-Aldrich), and DNase I (Roche) for 30 minutes at 37 °C with vigorous shaking. The cells were filtered through a 70 μm cell strainer and then subject to a Percoll^®^ gradient to reduce fibroblast contamination.

### Submerged and airlifting culture of CECs

Primary CECs were seeded on polyester Transwell® (Corning, Lowell, MA) membranes (0.4 μm pore size) at a density of 2 × 10^4^ cells/cm^2^ and cultured at 37 °C with 5% CO_2_. BEGM was added to both upper and lower compartments, and the inserts were kept at 37 °C with 5% CO_2_ until the cells reached confluence. Afterwards, medium was switched to a 1:1 mixture of BEGM and DMEM/F12 to induce stratification. Induction medium was added to both upper and lower compartments of the Transwell insert for submerged culture. Only the lower compartment had medium for airlifting culture, letting the cells on the membrane be exposed to air (Fig. 2C). The induction was kept for 1–2 weeks in the incubator.

### Spheroids formation and 3D culture of LGACs

Primary LGACs were suspended at a density of 1 × 10^7^ cells/mL in HepatoStim Medium (HSM), supplemented with 10 μg/mL epidermal growth factor (EGF) and 10% fetal bovine serum (FBS, Thermo Fisher Scientific). The suspension was added to a non-treated tissue culture flask (Thermo Fisher Scientific) to prevent adhesion. The flask was then placed on an orbital shaker at 100 rpm for 24 hours in the incubator (37 °C, 5% CO_2_). Cell spheroids were collected by centrifugation at 100 g for 5 minutes. The spheroids were resuspended with Matrigel® matrix (Corning; 10^8^ cells/mL of Matrigel). The mixture was added to TCP to form a thin layer of gel (100 μL/cm^2^) by incubating at 37 °C for 30 min. Images of spheroids were taken using Axio Imager 2 inverted fluorescent microscope (Carl Zeiss, Jena, Germany). The number and size of LG cell spheroids were analyzed using ImageJ software (National Institute of Health, NIH, Bethesda, MD). The spheroids were kept encapsulated in Matrigel for 1–2 weeks in the incubator.

### Coculture of CECs and LG cell spheroids

The coculture systems are diagramed in Figs 4A and 5A. LG cell spheroids were introduced into the system after CECs had undergone airlifting culture for 5 days. Three different designs were utilized: (I) No contact model: a thin layer of Matrigel with encapsulated LG cell spheroids was added to the bottom well of the Transwell system; (II) Indirect cell contact model: the LG cell spheroids were put on the other side of the insert membrane; therefore, the two types of cells were separated by the porous membrane; and (III) Direct cell contact model: Matrigel containing LG cell spheroids were layered on top of CECs on the periphery of the insert. All coculture systems were established in 12-well Transwell system, and 50 μL Matrigel was used in all coculture models. The seeding density for each cell type was the same as it was in monoculture. HSM medium was added to the lower compartment of the Transwell system only, and the cocultures were maintained for 1–2 weeks before harvest.

### Induction of dry eye-like inflammation with IL-1β

The direct cell contact coculture model was subject to IL-1β (Thermo Fisher Scientific; 10 ng/mL) exposure. The direct cell contact coculture model was constructed as described above, with the addition of IL-1β in the medium. Dexamethasone (10 μM; Sigma-Aldrich) was used as a treatment for IL-1β-induced inflammation. The cultures were maintained for one week, and the response of the direct cell contact model to the exposure of IL-1β, dexamethasone, and the two substances combined was analyzed. The results were also compared to monocultured samples under the same exposures.

### Reverse transcription and real-time quantitative polymerase chain reaction (RT-qPCR)

Total RNA was extracted and purified using RNeasy Mini Kit (Qiagen, Valencia, CA). cDNA synthesis was carried out with high capacity reverse transcription kit for RT-PCR (Thermo Fisher Scientific) according to the manufacturer’s protocol. Real-time PCR was performed on the StepOnePlus^™^ Real-Time PCR System using SYBR^®^ Green PCR Master Mix (Thermo Fisher Scientific). The relative expression level (fold change) of all targets was calculated by the ΔΔC_T_ method^39^ and normalized against the control samples with β-actin as the endogenous reference. Primer sequences are listed in Table 1.

**Table 1:** Primer sequences for RT-qPCR.

Gene		Sequence	
conjunctiva specific	*CK4*	forward	CAA CCT GAA GAC CAC CAA GA
		reverse	CAG AGT CTG GCA CTG CTT T
	*MUC5AC*	forward	CGC CTT CTT CAA CAC CTT CA
		reverse	TGG GCA AAC TTC TCG TTC TC
lacrimal gland specific	*AQP5*	forward	CAA CGC GCT CAA CAA CAA
		reverse	GTG AGT CGG TGG AAG AGA AA
	*LTF*	forward	GAT GCC ATG ACC CTG GAT AG
		reverse	GTC TGT GGC TTC GCT TCT
	*CDH1*	forward	CAC CAT CGC CAT GAG TCT T
		reverse	GAA TAA CCC AGT CCC TCT TCT G
inflammatory	*IL-6*	forward	GAA TAA TGA GAC CTG CCT GCT
		reverse	TTC TTC GTC ACT CCT GAA CTT G
	*IL-8*	forward	TGG ACC TCA CTG TGC AAA T
		reverse	GCT CAG CCC TCT TCA AGA AT
	*TNFα*	forward	GTA GTA GCA AAC CCG CAA GT
		reverse	GGT TGT CCG TGA GCT TCA T
	*MMP9*	forward	AGT ACC GAG AGA AAG CCT ACT T
		reverse	TGC AGG ATG TCA AAG CTC AC
internal control	*β-actin*	forward	GCT ATT TGG CGC TGG ACT T
		reverse	GCG GCT CGT AGC TCT TCT C

### Histology preparation

Cells cultured on TCP and Transwell membranes were fixed with 4% paraformaldehyde (PFA; Electron Microscopy Sciences, Hatfield, PA) and washed with PBS. The Transwell samples were further dehydrated through ethanol gradients, cleared in xylenes, and embedded in paraffin. Sections of 5 μm thick were used for histological stainings. Hematoxylin & eosin (H&E) and PAS staining kits (Sigma-Aldrich) were used according to the manufacturer’s manuals. Images of stained samples were taken on the Axio Imager 2 fluorescent microscope (Carl Zeiss).

### Immunohistochemistry

TCP and Transwell samples were first fixed with 4% PFA, followed by PBS rinse. F-actin was stained with Alexa Fluor® 647 phalloidin (Thermo Fisher Scientific) per manufacturer’s instructions. The nuclei were counterstained with 1 µg/mL DAPI (4′,6-diamidino-2-phenylindole dihydrochloride; Thermo Fisher Scientific). Staining with antibodies for the detection of specific antigens was performed as previously described^40^. Anti-rabbit mucin 5AC, lysozyme, and pan-cytokeratin monoclonal antibodies were purchased from Abcam (Cambridge, MA); Anti-rabbit cytokeratin 4 monoclonal antibody was purchased from Sigma-Aldrich. Axio Imager 2 fluorescent microscope (Carl Zeiss) and confocal laser scanning microscope 510 (LSM 510; Carl Zeiss) were used for imaging.

### β-Hexosaminidase assay

The secretion ability of LG cell spheroids was assessed by the measurement of NAG, a lysosomal enzyme in the tear fluid. The culture medium was replaced with DMEM/F12 and the cells were incubated at 37 °C for 2 hours. Carbamylcholine chloride (carbachol, Sigma-Aldrich; 100 μM) was added to the medium, and the samples were incubated for another 30 minutes. The media were collected and centrifuged at 200 g for 5 minutes. The resulting supernatants were analyzed for NAG catalytic activity with a NAG assay kit (Sigma-Aldrich). The absorbance at 405 nm or fluorescent intensity was measured using Synergy 2 microplate reader (BioTek, Winooski, VT) as previously described^18^. Total protein content in the samples was measured by Pierce BCA protein assay kit (Thermo Fisher Scientific).

### Mucin layer visualization by confocal microscopy

Mucins secreted by air-lifted samples were visualized and semi-quantified by loading Texas Red conjugated dextran solution (10 kDa MW, 2 mg/mL in PBS; Thermo Fisher Scientific) in the upper chamber of Transwell inserts (20 μL per 12-well insert), similar to previous studies^41, 42^. The Transwell cultures were gently rinsed with warm PBS to remove secreted mucins before the addition of Texas Red-dextran. The inserts were incubated for 24 hours to allow mucin secretion, and then examined using confocal microscopy with Z-stack scanning by LSM 510. The serial images were analyzed to generate 3D images for the measurement of mucin layer thin film with ZEN imaging software (Carl Zeiss) and ImageJ (NIH).

### Permeability tests with Dextran

Texas Red-dextran (10 kDa; 10 mg/mL in PBS) was used in the study. Before the test, medium in the lower compartment in the Transwell was switched to serum free DMEM/F12. Dextran solution was added in the insert and the Transwell cultures were incubated at 37 °C, 5% CO_2_ for 30 minutes. The concentration of Texas Red-dextran in both upper and lower compartments was calculated by measuring the fluorescence on Synergy 2 microplate reader (excitation: 520 nm; emission: 590 nm). A serial concentration of Texas Red-dextran solutions was made to construct the standard curve. Data is presented as the percentage of dextran molecules that diffused through the insert membrane.

### Statistical analysis

Experiments were run by at least triplets, and results are presented as mean values ± standard deviation. Data analysis was performed by GraphPad Prism, and the variance was analyzed by one-way ANOVA with post-hoc Tukey HSD for multiple comparison. A *P* value less than 0.05 was considered to be statistically significant.

## Electronic supplementary material

Supplementary information

## References

[1] Rolando M, Zierhut M (2001). The Ocular Surface and Tear Film and Their Dysfunction in Dry Eye Disease. Surv Ophthalmol.

[2] Lemp MA (2007). The definition and classification of dry eye disease: Report of the Definition and Classification Subcommittee of the international Dry Eye WorkShop (2007). Ocul Surf.

[3] Gipson IK (2007). The ocular surface: the challenge to enable and protect vision: the Friedenwald lecture. Invest Ophthalmol Vis Sci.

[4] Stern ME, Gao J, Siemasko KF, Beuerman RW, Pflugfelder SC (2004). The role of the lacrimal functional unit in the pathophysiology of dry eye. Exp Eye Res.

[5] Stern ME, Schaumburg CS, Pflugfelder SC (2013). Dry eye as a mucosal autoimmune disease. Int Rev Immunol.

[6] Stevenson W, Chauhan SK, Dana R (2012). Dry eye disease: an immune-mediated ocular surface disorder. Arch Ophthalmol.

[7] Gipson IK (2007). Research in dry eye: Report of the Research Subcommittee of the international dry eye WorkShop (2007). Ocul Surf.

[8] Smith JA (2007). The epidemiology of dry eye disease: Report of the Epidemiology Subcommittee of the international Dry Eye WorkShop (2007). Ocul Surf.

[9] Huh D (2010). Reconstituting organ-level lung functions on a chip. Science.

[10] Kim HJ, Ingber DE (2013). Gut-on-a-Chip microenvironment induces human intestinal cells to undergo villus differentiation. Integr Biol (Camb).

[11] Torisawa YS (2014). Bone marrow-on-a-chip replicates hematopoietic niche physiology *in vitro*. Nat Methods.

[12] Huh D (2012). A human disease model of drug toxicity-induced pulmonary edema in a lung-on-a-chip microdevice. Sci Transl Med.

[13] Zhong X (2014). Generation of three-dimensional retinal tissue with functional photoreceptors from human iPSCs. Nat Commun.

[14] McUsic AC, Lamba DA, Reh TA (2012). Guiding the morphogenesis of dissociated newborn mouse retinal cells and hES cell-derived retinal cells by soft lithography-patterned microchannel PLGA scaffolds. Biomaterials.

[15] Chan YK (2015). *In Vitro* Modeling of Emulsification of Silicone Oil as Intraocular Tamponade Using Microengineered Eye-on-a-Chip. Invest Ophthalmol Vis Sci.

[16] Puleo CM, McIntosh Ambrose W, Takezawa T, Elisseeff J, Wang TH (2009). Integration and application of vitrified collagen in multilayered microfluidic devices for corneal microtissue culture. Lab Chip.

[17] Chung SH, Lee JH, Yoon JH, Lee HK, Seo KY (2007). Multi-layered culture of primary human conjunctival epithelial cells producing MUC5AC. Exp Eye Res.

[18] Lin H (2016). Three-Dimensional Culture of Functional Adult Rabbit Lacrimal Gland Epithelial Cells on Decellularized Scaffold. Tissue Eng Part A.

[19] Spaniol K (2015). Engineering of a Secretory Active Three-Dimensional Lacrimal Gland Construct on the Basis of Decellularized Lacrimal Gland Tissue. Tissue Eng Part A.

[20] Hirayama M (2013). Functional lacrimal gland regeneration by transplantation of a bioengineered organ germ. Nat Commun.

[21] Tiwari S (2012). Establishing human lacrimal gland cultures with secretory function. PLoS One.

[22] Ding C, Lu M, Huang J (2011). Changes of the ocular surface and aquaporins in the lacrimal glands of rabbits during pregnancy. Mol Vis.

[23] Goers, L., Freemont, P. & Polizzi, K. M. Co-culture systems and technologies: taking synthetic biology to the next level. *J R Soc Interface* **11**, doi:10.1098/rsif.2014.0065 (2014). 10.1098/rsif.2014.0065 PMC4032528 24829281

[24] Wallace CS, Truskey GA (2010). Direct-contact co-culture between smooth muscle and endothelial cells inhibits TNF-alpha-mediated endothelial cell activation. Am J Physiol Heart Circ Physiol.

[25] Dinarello CA (2002). The IL-1 family and inflammatory diseases. Clin Exp Rheumatol.

[26] Nagelhout TJ, Gamache DA, Roberts L, Brady MT, Yanni JM (2005). Preservation of tear film integrity and inhibition of corneal injury by dexamethasone in a rabbit model of lacrimal gland inflammation-induced dry eye. J Ocul Pharmacol Ther.

[27] Viau S (2008). Time course of ocular surface and lacrimal gland changes in a new scopolamine-induced dry eye model. Graefes Arch Clin Exp Ophthalmol.

[28] Argueso P (2002). Decreased levels of the goblet cell mucin MUC5AC in tears of patients with Sjogren syndrome. Invest Ophthalmol Vis Sci.

[29] Shatos MA (2003). Isolation and characterization of cultured human conjunctival goblet cells. Invest Ophthalmol Vis Sci.

[30] Gipson IK (2016). Goblet cells of the conjunctiva: A review of recent findings. Prog Retin Eye Res.

[31] Ang LP (2010). Cultivated human conjunctival epithelial transplantation for total limbal stem cell deficiency. Invest Ophthalmol Vis Sci.

[32] Spurr-Michaud SJ, Gipson IK (2013). Methods for culture of human corneal and conjunctival epithelia. Methods Mol Biol.

[33] Schrader S, Kremling C, Klinger M, Laqua H, Geerling G (2009). Cultivation of lacrimal gland acinar cells in a microgravity environment. Br J Ophthalmol.

[34] Schechter J (2002). Growth of purified lacrimal acinar cells in Matrigel raft cultures. Exp Eye Res.

[35] Kleinman HK, Martin GR (2005). Matrigel: basement membrane matrix with biological activity. Semin Cancer Biol.

[36] Hann LE, Tatro JB, Sullivan DA (1989). Morphology and function of lacrimal gland acinar cells in primary culture. Invest Ophthalmol Vis Sci.

[37] Meneray MA, Fields TY, Bromberg BB, Moses RL (1994). Morphology and physiologic responsiveness of cultured rabbit lacrimal acini. Invest Ophthalmol Vis Sci.

[38] Selvam S (2007). Tissue-engineered tear secretory system: functional lacrimal gland acinar cells cultured on matrix protein-coated substrata. J Biomed Mater Res B Appl Biomater.

[39] Livak KJ, Schmittgen TD (2001). Analysis of relative gene expression data using real-time quantitative PCR and the 2(−Delta Delta C(T)) Method. Methods.

[40] Zhou H (2014). Vitrified collagen-based conjunctival equivalent for ocular surface reconstruction. Biomaterials.

[41] Worthington EN, Tarran R (2011). Methods for ASL measurements and mucus transport rates in cell cultures. Methods Mol Biol.

[42] Lee H (2014). DA-6034-induced mucin secretion via Ca2+-dependent pathways through P2Y receptor stimulation. Invest Ophthalmol Vis Sci.

