# Supplementary material for: An In Vitro Model for the Ocular Surface and Tear Film System
Source: Sci Rep. 2017 Jul 21;7:6163. doi: 10.1038/s41598-017-06369-8 (PMC5522434; doi:10.1038/s41598-017-06369-8)

**An *In Vitro* Model for the Ocular Surface and Tear Film System**

Qiaozhi Lu^1,2^, Hongbo Yin^3^, Michael P. Grant^4*^ and Jennifer H. Elisseeff^1,5*^

1. Translational Tissue Engineering Center, Wilmer Eye Institute, Johns Hopkins School of Medicine, Baltimore, MD 21231
2. Department of Materials Science and Engineering, Johns Hopkins University, Baltimore, MD 21218
3. Department of Ophthalmology, West China Hospital, Sichuan University, Chengdu 610041, China
4. Oculoplastics Division, Ocular and Orbital Trauma Center, Wilmer Eye Institute, Johns Hopkins School of Medicine, Baltimore, MD, 21287
5. Department of Biomedical Engineering, Johns Hopkins University, Baltimore, MD 21231

*Corresponding authors:

Michael P Grant, MD PhD (600 N. Wolfe St. Baltimore, MD 21287; phone: 410-955-1112; email: mgrant@jhmi.edu); Jennifer H Elisseeff, PhD (400 N. Broadway Baltimore, MD 21231; phone: 410-614-6837; email: jhe@jhu.edu)

**Supplementary Materials and Methods**

1. **Enrichment of goblet cells with Percoll density gradient**

In order to increase the density of goblet cells, the primary cell suspension was layered on a Percoll^®^/PBS (Sigma-Aldrich) density gradient which ranged from 18% to 57% (v/v; Fig. S1A), and centrifuged for 10 minutes at 100 g with zero acceleration or deceleration. Based on the results of gene expression study (Fig. S1B) of the cells from each layer, 30% Percoll/PBS was chosen for further study. The top and bottom cell layers (Fig. S1A) and unseparated CECs were collected and seeded on tissue culture plates (TCP), at a density of 2×10^4^ cells/cm^2^, and cultured at 37°C with 5% CO_2_, with Bronchial Epithelial Cell Growth Medium (BEGM™, with all supplements added; Lonza, Walkersville, MD).

**Supplementary Figures and Figure Legends**

Figure S1. Enrichment of goblet cell density within primary conjunctival epithelial cell populations by Percoll density gradient centrifugation

(A) Primary conjunctival epithelial cells were subject to a Percoll density gradient and the centrifugation resulted in six cell populations; the percent (v/v) of Percoll in each layer was shown; (B) Phenotypic analysis of each cell population by gene expression (RT-qPCR); **p* < 0.05; ***p* < 0.001.


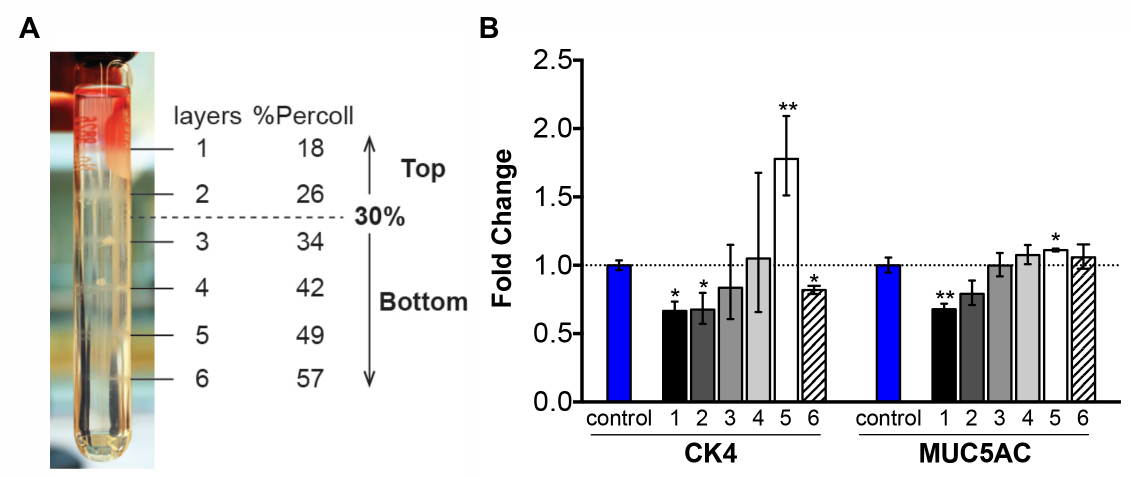


Figure S2. Size distribution of lacrimal gland spheroids

(A) A typical image of lacrimal gland spheroids. Five similar images were used for size analysis by Image J) Scale bar 400 μm. (B) Size distribution histogram of lacrimal gland spheroids. The average size is 124 ± 19 µm.


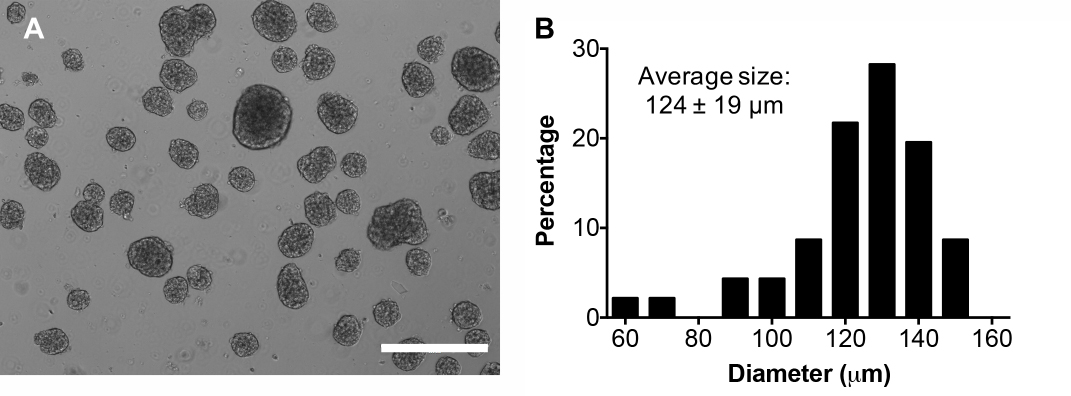


Figure S3. Influence of IL-1β addition in the coculture system on gene expression at early time point (six hours)

(A-C) Gene expression of the coculture system after the addition of IL-1β. (A) Inflammatory genes; (B) conjunctival epithelial specific genes; (C) lacrimal gland specific genes. **p* < 0.05; ***p* < 0.001. Significance indicates comparison with the control group if not stated otherwise.


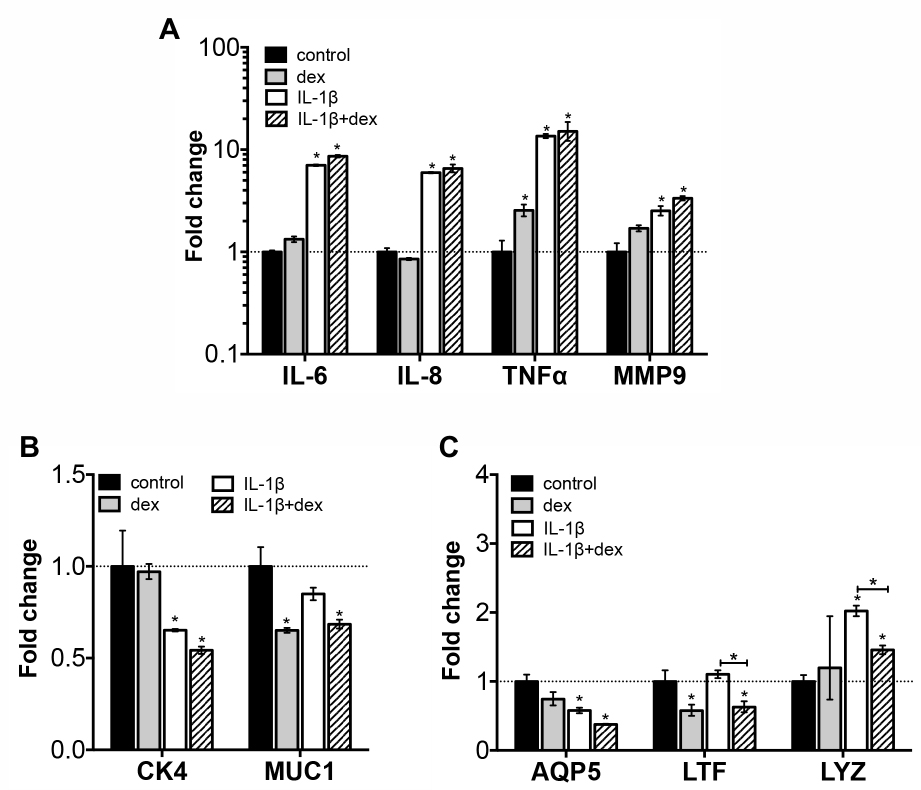


Figure S4. Responses of cells under monoculture after cytokine IL-1β stimulation.

Gene expression of monocultured LG spheroids and CECs when exposed to IL-1β. (A) Proinflammatory factors; (B) Lacrimal gland specific genes; (C) Conjunctival epithelial specific genes. Dex: dexamethasone, used as a treatment to inflammatory caused by IL-1β. *p < 0.05; **p < 0.001. Significance indicates comparison with the control group if not stated otherwise.


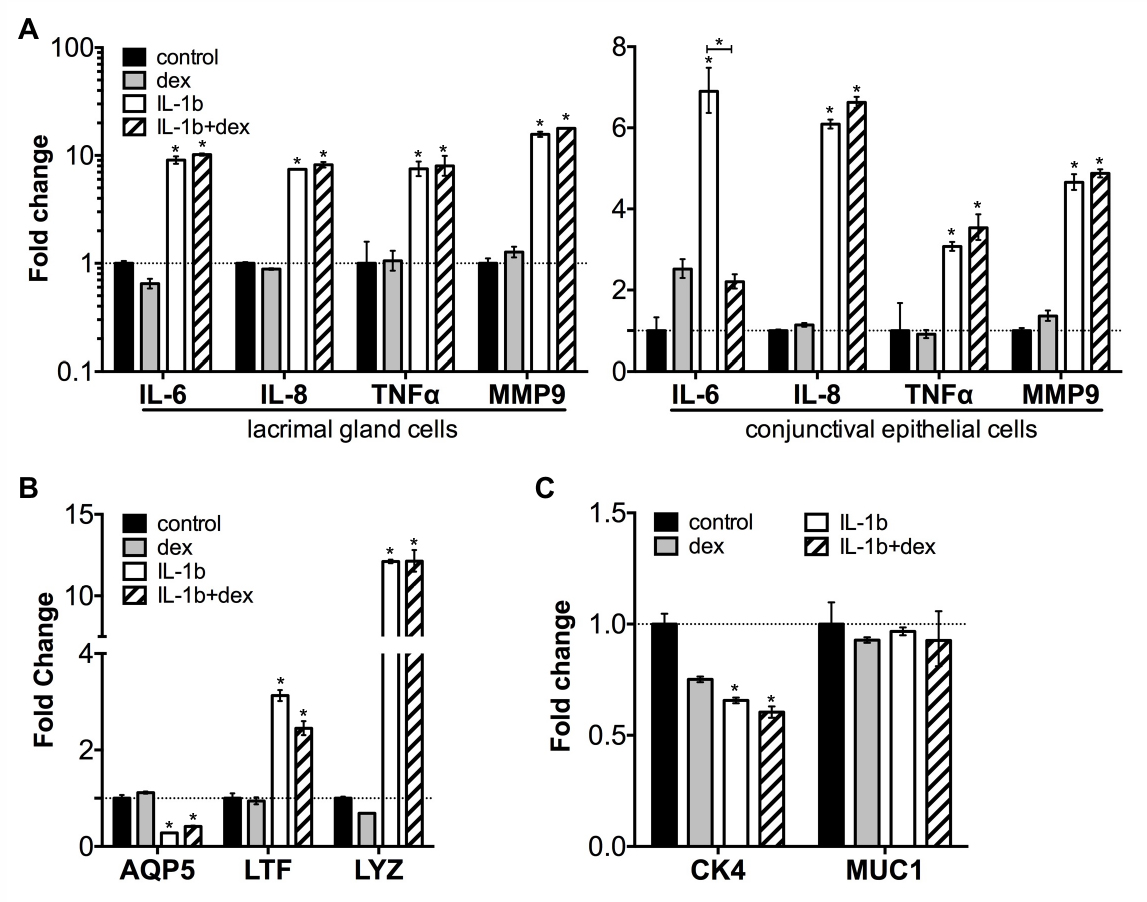

Supplement: Supplementary file 1 — Supplementary information [file 41598_2017_6369_MOESM1_ESM.docx]
